# Deficiency of Interleukin-1 Receptor Antagonist (DIRA): Report of the First Indian Patient and a Novel Deletion Affecting *IL1RN*

**DOI:** 10.1186/1546-0096-13-S1-P143

**Published:** 2015-09-28

**Authors:** LO Mendonca, L Malle, FX Donavan, SC Chandrasekharappa, GA Montealegre, D Chapelle, D Suri, R Goldbach-Mansky, AA de Jesus

**Affiliations:** 1 University of Sao Paulo, Clinical Immunology and Allergy, Sao Paulo, Brazil; 2 National Institute of Artrhitis and muskuloeskeletal and skin disease, Translational autoinflammatory disease, Bethesda, USA; 3 National Human Genome Research Institute, Genomics Core, Bethesda, USA; 4 Postgraduate Institute of Medical Education and Research, Department of Pediatrics, Chandigarh, India

## Introduction

Deficiency of interleukin-1-receptor antagonist (DIRA) is a rare autoinflammatory disease clinically characterized by early-onset generalized pustulosis, multifocal osteomyelitis and elevation of acute-phase reactants. DIRA is caused by autosomal recessive loss of function mutations in *IL1RN*. Seven DIRA causing mutations have been described, including one 175Kb genomic deletion, 4 premature stopcodons, 1 inframe deletion, and 1 missense mutation.

## Objective

To report a novel disease-causing deletion affecting *IL1RN* gene in an Indian patient with DIRA.

## Methods

The patient was enrolled into a NIH natural history protocol. Peripheral blood genomic DNA was obtained and patient was genotyped for the detection of copy number variations (CNVs) by a SNP array technique. As determined by SNP array, primers flanking the deletion ends were designed and the breakpoint area was amplified by polymerase chain reaction (PCR) and sequenced by Sanger technique.

## Results

Clinical description: A 5 month-old Indian girl, born to healthy non-consanguineous parents presented with pain on manipulation and irritability at the 3^rd^ week of life and developed a mild pustular rash limited to the back of neck and upper forehead. Bone scintigraphy suggested osteomyelitis of multiple bones, including ribs, clavicles and long bones and cultures from left wrist bone biopsy material were negative. Primary immunodeficiency work up was negative. There was no clinical response to a 5-week course of broad-spectrum antibiotics, and thus an autoinflammatory condition was suspected. Prednisolone was initiated and the patient had significant clinical improvement. Due to a clinical suspicion of DIRA, the patient was enrolled in a NIH protocol and started on recombinant interleukin-1 receptor antagonist (anakinra). Anakinra initiation resulted in marked and sustained clinical and laboratory improvement.

Genetic analysis: SNP array analysis of patient's genomic DNA showed loss of heterozygosity for all SNPs in a region of approximately 21.4 to 23.7kb, indicating a homozygous deletion. PCR and sequencing of the breakpoint area allowed us to identify the breakpoints of the deletion, at chr2_hg19_113,865,011 and chr2_hg19_113,887,227, confirming a homozygous 22,216bp deletion that spans the first four exons of *IL1RN* (NM_173843). This deletion has not been previously reported in patients with DIRA.

## Conclusion

We describe the first Indian patient with DIRA and a novel homozygous 22Kb deletion spanning 2 coding exons of IL1RN. The primers designed to detect the novel deletion will be useful to screen Indian patients with a clinical suspicion of DIRA.

